# Assessment of myocardial strain in patients with myocarditis by cardiac magnetic resonance imaging

**DOI:** 10.1186/1532-429X-16-S1-P314

**Published:** 2014-01-16

**Authors:** Florian Andre, Florian T Stock, Kristin Breuninger, Fabian aus dem Siepen, Evangelos Giannitsis, Grigorios Korosoglou, Hugo A Katus, Sebastian Buss

**Affiliations:** 1 Department of Cardiology, University of Heidelberg, Heidelberg, Germany

## Background

Myocarditis is associated with a considerable morbidity and mortality in the acute phase as well as in the long term. It is found in up to 12% of young adults with sudden cardiac death and is regarded as a cause of dilated cardiomyopathy which is currently the most frequent reason for heart transplantation. Therefore, techniques for the diagnosis and the assessment of prognosis are of great interest. In this study we evaluate the novel post-procession feature tracking imaging (FTI) algorithm for strain analysis on patients with myocarditis.

## Methods

We retrospectively included 36 patients (31 male, 5 female) who were admitted with acute myocarditis. A control group (31 male, 5 female) was drawn from a reference population of proven healthy volunteers and was matched with regard to age and gender. CMR imaging was performed on a 1.5 T whole-body MRI (Achieva, Philips Healthcare). Short axis views covering both ventricles as well as 2-, 3- and 4-chamber views were obtained using a SSFP sequence. Enddiastolic and endsystolic volumes as well as ejection fraction (EF) were derived from short axis segmentation. In addition we measured the circumferential und longitudinal strain applying a post-procession FTI algorithm (TomTec Imaging Systems).

## Results

The study population and the control group showed similar characteristics regarding age and gender (40.3 ± 13.7 yrs. vs. 40.3 ± 15.7 yrs., p > 0.99). In patients with myocarditis the EF was significantly reduced compared to healthy controls (54.3 ± 8.4% vs. 67.8 ± 5.3%, p < 0.001). Furthermore the patients showed significantly lower values for the global circumferential strain (-24.4 ± 4.2% vs. -28.8 ± 3.8%, p < 0.001) as well as for the global longitudinal strain (-17.7 ± 4.5% vs. -23.6 ± 3.0%, p < 0.001). Global circumferential strain (r = -0.77, p < 0.001) and global longitudinal strain (r = -0.65, p < 0.001) correlated well with EF. In the subgroup of myocarditis patients with preserved ejection fraction (EF≥55%, 16 pts.) the global longitudinal strain (-20.4 ± 4.5% vs. -23.7 ± 2.6%, p < 0.05) was significantly reduced compared to the age- and gender matched control subgroup whereas the global circumferential strain did not show a significant difference (-27.3 ± 2.7% vs. -28.9 ± 3.8%, p = n.s.).

## Conclusions

FTI strain analysis offers a fast quantitative assessment of myocardial strain patterns without the need for additional dedicated strain imaging sequences. Myocarditis patients with preserved EF show reduced longitudinal strain whereas the circumferential strain is not significantly impaired. Further investigations may address the impact of this finding on clinical long term outcome.

## Funding

None.

**Figure 1:**
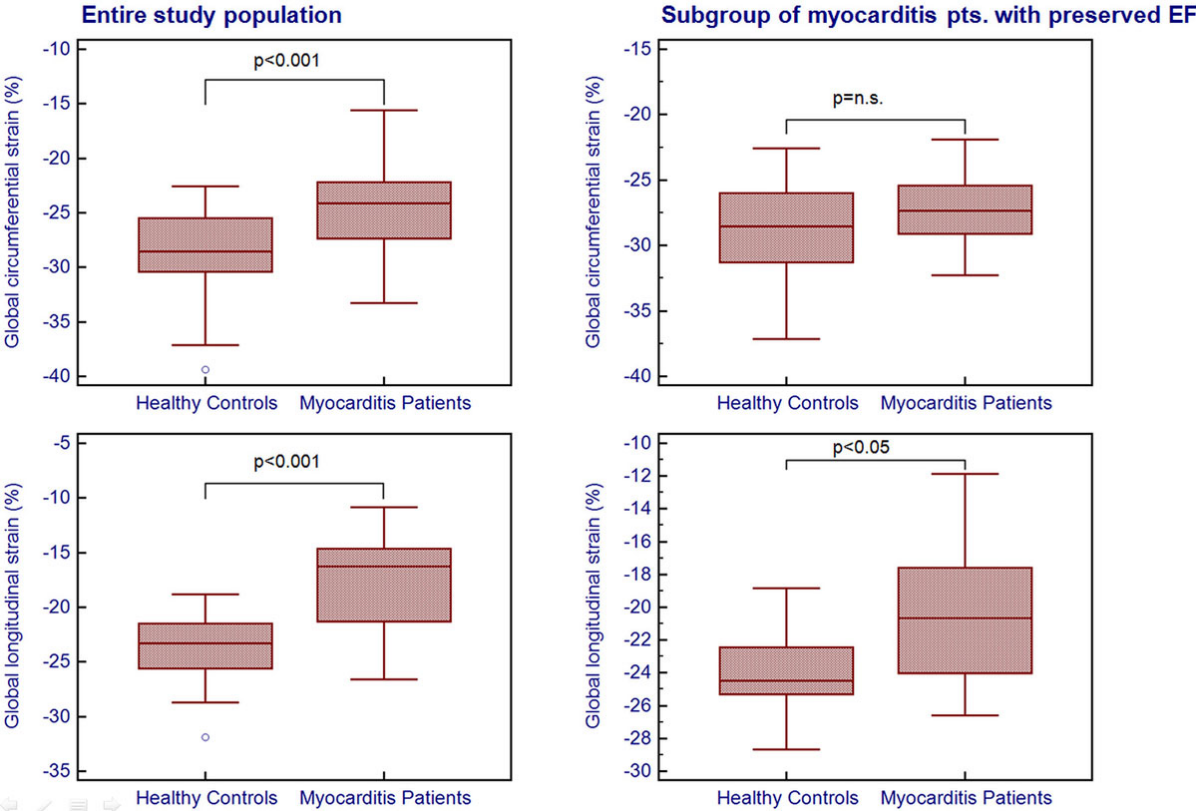
**Comparison of circumferentaial and longitudinal**. pts: patients EF: ejection fraction.

